# Computer vision based individual fish identification using skin dot pattern

**DOI:** 10.1038/s41598-021-96476-4

**Published:** 2021-08-19

**Authors:** Petr Cisar, Dinara Bekkozhayeva, Oleksandr Movchan, Mohammadmehdi Saberioon, Rudolf Schraml

**Affiliations:** 1 Laboratory of Signal and Image Processing, Institute of Complex Systems, FFPW, CENAKVA, University of South Bohemia in Ceske Budejovice, Zámek 136, Nové Hrady, 373 33 Czech Republic; 2 Wavelab, University of Salzburg, Salzburg, Austria

**Keywords:** Image processing, Ichthyology

## Abstract

Precision fish farming is an emerging concept in aquaculture research and industry, which combines new technologies and data processing methods to enable data-based decision making in fish farming. The concept is based on the automated monitoring of fish, infrastructure, and the environment ideally by contactless methods. The identification of individual fish of the same species within the cultivated group is critical for individualized treatment, biomass estimation and fish state determination. A few studies have shown that fish body patterns can be used for individual identification, but no system for the automation of this exists. We introduced a methodology for fully automatic Atlantic salmon (*Salmo salar*) individual identification according to the dot patterns on the skin. The method was tested for 328 individuals, with identification accuracy of 100%. We also studied the long-term stability of the patterns (aging) for individual identification over a period of 6 months. The identification accuracy was 100% for 30 fish (out of water images). The methodology can be adapted to any fish species with dot skin patterns. We proved that the methodology can be used as a non-invasive substitute for invasive fish tagging. The non-invasive fish identification opens new posiblities to maintain the fish individually and not as a fish school which is impossible with current invasive fish tagging.

## Introduction

The trend toward increased automation in agriculture is significant^1^ and the aquaculture sector is not exception. The development of new methods for machine vision and digital cameras enables the automation of several asks under the non-trivial conditions of fish cultivation^2^. The main idea of the automatized aquaculture concept is implementation of precision fish farming^3^ (The automation of aquaculture production improves the control, monitoring and documentation of the biological process of fish growth. Based on automatically extracted knowledge, the farmers can increase the profits by controlling diseases and monitor fish welfare and fish growth. Early disease detection and the prediction of outbreaks can safeguar the livestock of fish^4^. One of the critical components of automation is individual fish identification^5^ (Yusup et al., 2020).

The standard approach to the identification of individual fish of the same specie is tagging^6^. There are several disadvantages and limitations of this invasive approach: the high mortality after injection in specific cases^7,8^ (Bolland et all., 2009; McMahon et al., 1996), stress caused the fish by the application of the invasive approach, need for the fish to be caught for identification, time-consuming nature of the method^9^ (Whitfield et al., 2004), and limited fish size. The possibility of the non-invasive identification of individuals becomes an alternative for fish tagging^10^ and address all the previously listed disadvantages.

Today, photo- or video-based identification systems are used in agriculture^11^ and wildlife for ensuring population welfare and estimating the size of the population^12,13^. All systems use the visible pattern of the animal for the identification of the species or individuals of the same species. The identification principle is based on human biometric identification as documentefor cow iris-based identification^14^. Similar issues of aging^15^ are therefore recoginized in animal identification.

In the aquaculture sector, machine vision is usually used to identify fish species^16^. Several studies^17–23^, exist on the non-invasive individual identification of fish using images but only few of them use machine vision. The majority of the studies use human experts for identification based on the fish images^20,22^. These studies proved that fish appearance based individual identification is feasible for the carp (*Ciprinus carpio*) (15 fish, 95.76% accuracy)^20^ and catshark (*Scyliorhinus canicular*) (25 fish, 99.6% accuracy)^22^ in the short-term. A long-term study of wild populations of cutthroat trout^21^, in which datasets from 1997 and 1999 were compared, showed that two adult fish can be identified using the skin spots after two years. The stability of the pattern was also proved by Stien^18^. They manually labeled the dots on salmon and performed the identification of 25 fish for ten months. The identification accuracy was 85%.

Only a few papers have presented results for semi-automatized identification, with a minimal number of fish^17,19^ and it has been tested only for short-term periods.

A computer-assisted approach for the identification of 30 individuals was used for armored catfish^17^. Computer-assited means that the 20 most similar images were ranked based on the SIFT (scale-invariant feature transform). The humans used this ranked list for identification. The identification accuracy was 99% for the images taken on two days.

The only fully automated approach has been described by Al-Jubouri^19^, who performed the identification of five zebrafish (*Danio rerio*) with 99% accuracy. The histogram of the hue-saturation-value color space of the part of zebrafish stripes was coupled with the KNN (*K*-nearest neighbour) classifier. All images were collected for one day.

To the best of our knowledge, there is no study using fully automated computer vision for individual fish identification working with a high number of fish or tested for a long-term period. The abovementioned studies showed the feasibility of appearance-based identification but for a limited number of fish or species with low value for the aquaculture industry.

Therefore, this study’s aim goes beyond the mentioned studies and introduces a fully automatic method for the long-term individual identification of the commercially important species, Atlantic salmon (*Salmo Salar*).

## Methods

### Experimental animal

The Atlantic salmon (*Salmo salar*), one of the most economically important fish species, was used in the study. The experiment was conducted at the NOFIMA experimental infrastructure in Sunndalsora, Norway. A total of 328 farmed Atlantic Salmon were used in the experiment. The average fish weight was 251 ± 21 g and the length 29.5 ± 2.5 cm. The age of the fish was 5 months. The 328 fish were used for short-term identification to test the identification power of the pattern. Thirty of the fish were tagged with PIT (passive integrated transponder) tags and used for long term identification to test pattern stability. The tagged fish were cultivated in a 2m^3^ recirculation freshwater tank for six months. The fish were only manipulated for data acquisition. The experimental procedure followed the standard operational procedures and regulations of NOFIMA. The experimental protocol was approved by the Norwegian Animal Research Authority and by the ethical advisor of the AQUAEXCEL^2020^ project. The study is in accordance with ARRIVE guidelines.

### Experimental design

**Figure 1:**
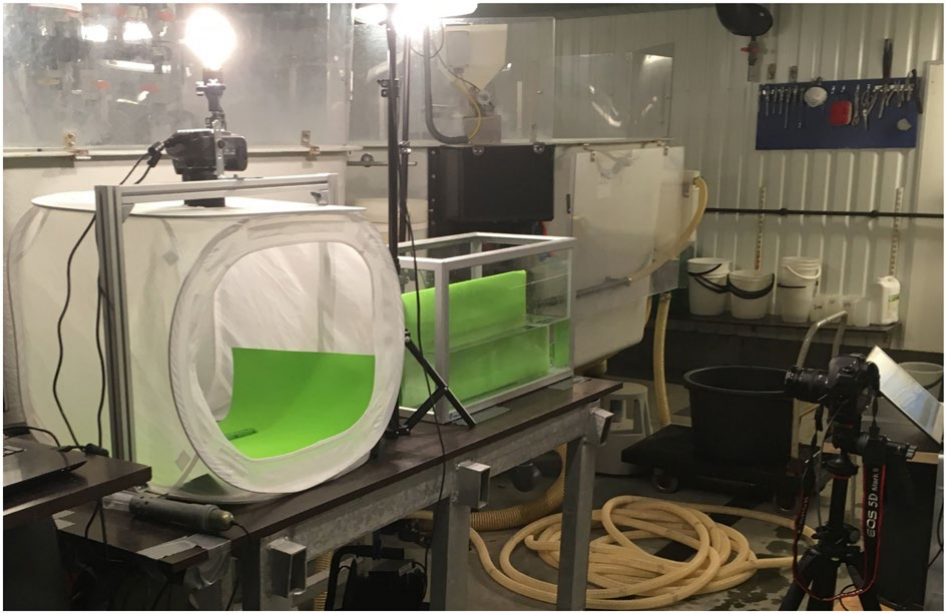
Experimental setup for data collection. Photo tent with camera installed on top, aquarium with the camera installed in front of it, and LED lights for scene illumination. The green background was installed in the tent and aquarium.

**Figure 2:**
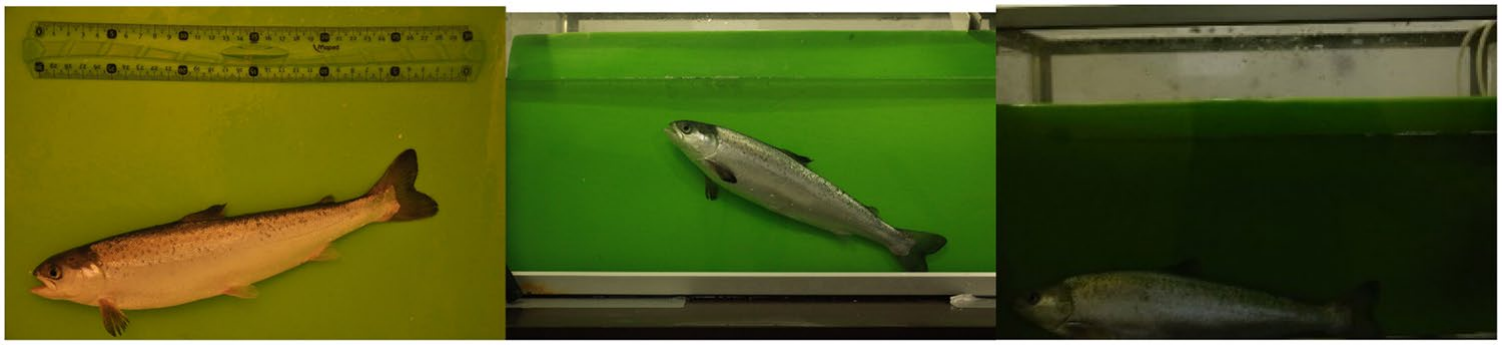
Example of images from data collection. Left—tent data, middle—aquarium data SL1, right—aquarium data SL3. Middle and right images show the different illumination conditions for data collection.

A total of 4 data collections (session—s) were performed over 6 months at 2 months intervals. Two types of data were taken in each session: lateral view images of the fish out of the water (in a photographic tent; see Fig. 1) and underwater (in a small aquarium; see Fig. 1). During each session, the fish were caught in the tank, anesthetized (FINQUEL MS-222) and moved to a single-layer white fabric photographic tent with controlled illumination and a foamy green background. The photographic tent was used to ensure a uniform light intensity over the fish skin. The foamy green background allowed for easier background segmentation in the image pre-processing phase. The scene was illuminated by LED bulb light to control the light conditions in the tent. Only the left sides of the fish were photographed. The NIKON D90 digital camera was used to take approximately eight images of each fish in RAW format. The fish were moved and rotated (± 45 degrees) in the tent to take pictures of the fish in different positions and rotations (Fig. 2). The resolution was 4288 × 2848 pixels, with 12 bits/pixel, and three color channels (i.e., red, green and blue). After data collection in the tent, the fish were moved into an aquarium with dimensions of 60 × 35 × 30 cm. The illumination was changed during data collection to simulate real production conditions. The Canon EOS 5D Mark II digital camera was used to take approximately eight images of each fish in aquarium. The fish were moved and rotated (± 45 degrees) in the aquarium to take their pictures in different positions and rotations (Fig. 2). The resolution was 5616 × 3744 pixels, with 12 bits/pixel and three color channels. Both cameras were set to the automatic mode. After data collection, the fish were moved back to the cultivati tank for recovery. All the fish fully recovered.

**Figure 3:**
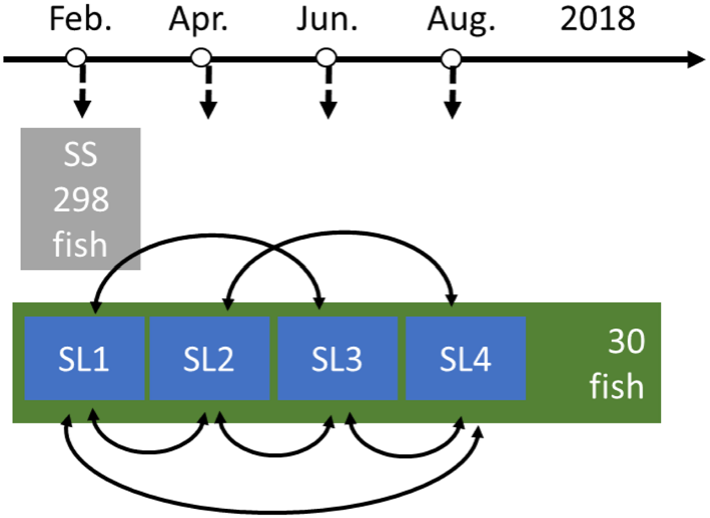
Visualization of data collection sessions. SS—298 fish data collection, SL1, SL2, SL3, SL4—30 tagged fish data collection over the time. The arrows between the SL datasets indicate the identification tasks performed for all combinations of the datasets.

The first data collection session was different from the subsequent three sessions. Images (tent and aquarium) were collected for 328 fish in the first data collection for five days. Of these, 298 fish were not tagged, and their images were collected only in the first session. This image dataset is marked SS (see Fig. 3). Images of the 30 tagged fish were also collected as the long-term dataset during the next six months with two months intervals. The first dataset is marked SL1 and the rest are numbered according to their session (see Fig. 3).

## Identification design

Two specific identification tasks were performed with the recorded datasets. The first focused on testing the uniqueness of the skin dot pattern for individual identification and is called short-term. The identification was performed for the fish images collected for five days. The SS dataset (298 fish), together with SL1 dataset (30 fish), was used for this task. The images for each fish in both datasets were divided randomly into templates and test images. The templates were used as the representative images of fish, and the test images were used for the identification. All fish were compared with all the others within the SS and SL1 datasets. In total, 328 fish were used for short-term identification.

The second identification task was focused on the testing of the long-term stability of the patterns for identification. The datasets SL1, SL2, SL3 and SL4 were used for this task. Two identifications were performed for these datasets. The within dataset identification was performed for each dataset separately. The same procedure as used for the SS dataset was used. This task tested the identification within the S1, S2, S3 and S4 datasets independently. The results of the within-datasetidentification show the identification accuracy according to fish age. The more important task was to test the long-term stability of the patterns. Therefore, the identification was performed with the combinations of all four datasets. This task was to test if the pattern was stable over six months of fish cultivation. All possible combinations of the datasets were used for identification (see Fig. 3).

## ROI selection

Before the images in the dataset were processed, they were manually checked to remove images corrupted during data collection. The number of images per fish varied from 5 to 9.

**Figure 4:**
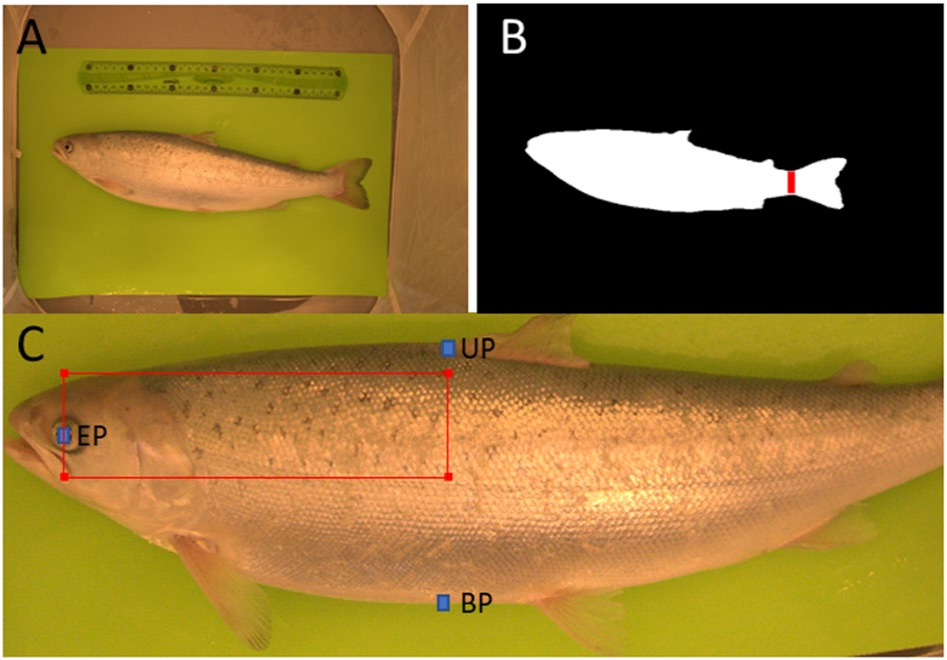
Process of ROI localization. **A** Original image of the fish in the tent. **B** Localized objective image of the fish with the narrowest area before the tail fin. **C** Fish with localized points EP (eye position), UP (upper fin beginning) and BP (belly point at the vertical position of UP); red rectangle represents the ROI.

The regions of interest (ROIs) were automatically extracted for all images in all datasets using image processing methods. The green background was used for the segmentation of the fish and background. First, the area with the specific colour (green) was detected based on the known hue and saturation values of the background in terms of hue, saturation and lightness colour space. Thresholding was used for background detection. The second step was to localize the fish within the background area. The fish was detected as the largest object inside the background area. The detected fish object is shown in Fig. 4. The object was rotated using an estimated ellipse around the object (Matlab R2020b – function regionprops (image,'Orientation')) to compensate for rotation of the fish in the image. Because the fish tail fin is semi-transparent and the segmentation of this part is not stable, the narrowest area of the fish before the tail fin was detected (see Fig. 4B). The length of the fish was then defined from the head tip to the narrowest area. The approximate area of the upper fin was estimated to be in the region of 3/8 to 5/8 of the length of the detected object (without the tail fin). This area was used to search for the beginning of the upper fin. The segmented fish border of the area was divided into the left and right half.

**Figure 5:**
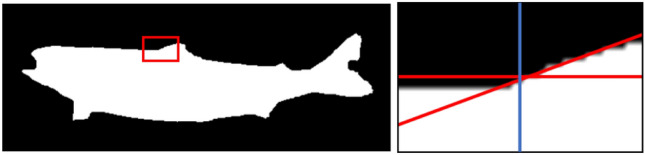
Localization of the upper fin beginning. Left—Segmented fish with selected region of upper fin beginning. Right—Magnification of selected region; blue line—horizontal center of the image, red lines—lines fitted to the left and right halves of the border pixels. Produced by Matlab R2020b.

The line was fitted to the left and right pixesls and the intersection was taken as the beginning of the upper fin (see the example in Fig. 5). The localized upper fin position is marked UP.

The approximate area of fish eye was estimated between 0 and 1/3 of the UP position (in horizontal axes). The exact area of the fish eye was detected by the thresholding of the grayscale image of the selected area. All pixels with intensitys lower than 20 (the threshold was determined experimentally and works for all datasets) were marked as eye pixels. The eye position EP was calculated as the centroid of the eye pixels.

The belly point (BP), which represents the height of the fish at the horizontal position of UP, was detected as the point at the border of the fish belly. This point wass used to determine the height of the ROI.

Based on the determined points UP, BP and EP, the ROI was selected in the image. The ROI wass selected as the area between EP and UP in the horizontal direction and between UP + (BP-UP)/20 and BP/2 in the vertical direction. See an example of a ROI in Fig. 4.

## DOT based approach

**Figure 6:**
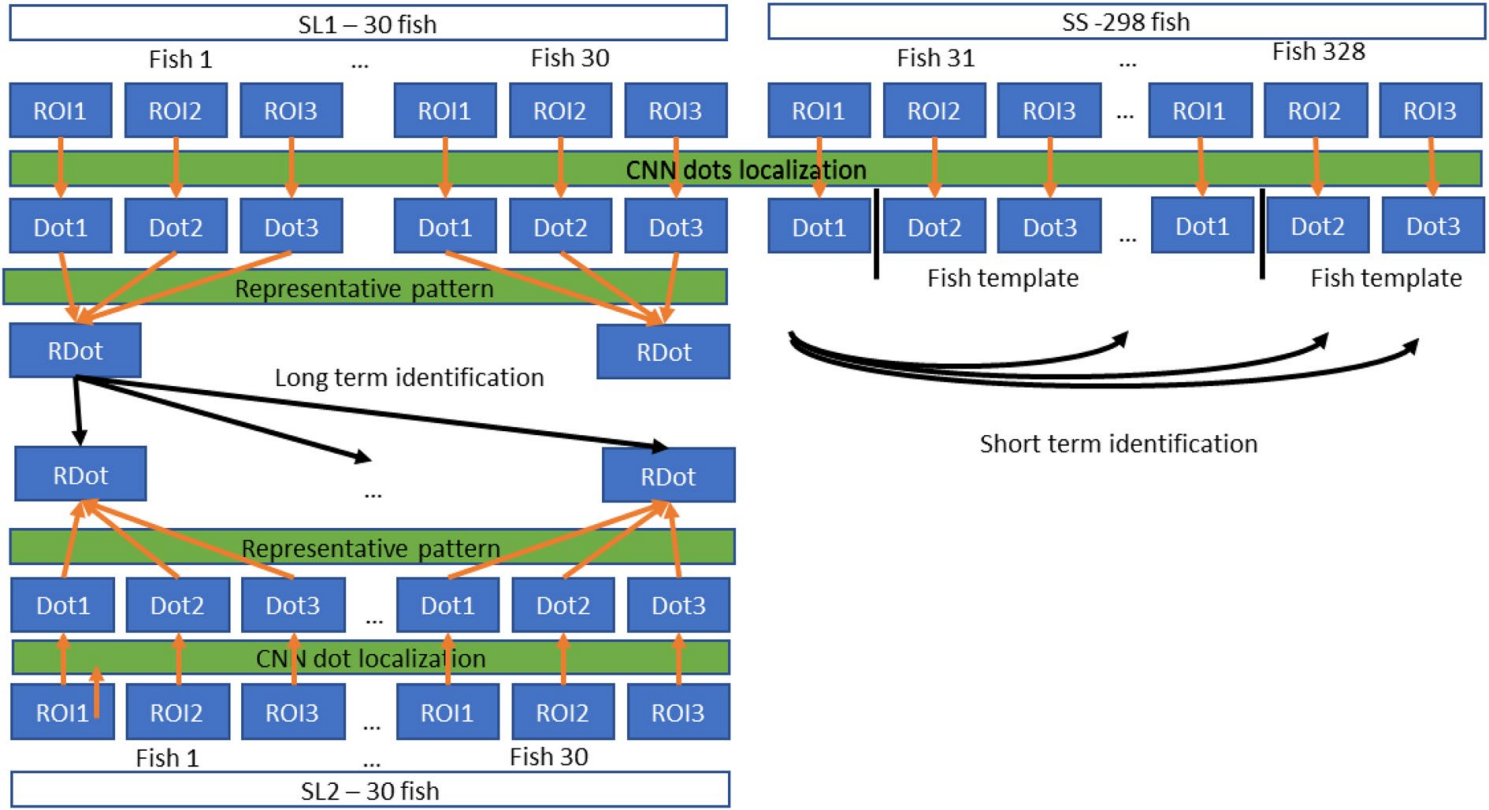
E Scheme of dot localization-based approach to identification. The dots (Dot) for each ROI of each fish are detected using CNN. The dots are used for short-term identification, where two images are used as the pattern and one image as an unknown image. The representative dot pattern (RDot) is calculated for each fish from all fish dot patterns. The representative dot pattern is used for long-term identification for the SL1, SL2, SL3 and SL4 datasets.

Identification based on the dots’ exact position on the fish skin consists of several steps (see Fig. 6). First, the dots in the normalized ROI are localized using convolutional neural network (CNN) for all images of the particular fish. The localized dots are used for the identification of individuals for the SS dataset. Then the representative dot pattern is created as a subset of the detected dots for the particular fish from the images of that fish. The representative dot pattern is used for the identification of individuals for the SL datasets.

### DOTs localization

**Figure 7:**
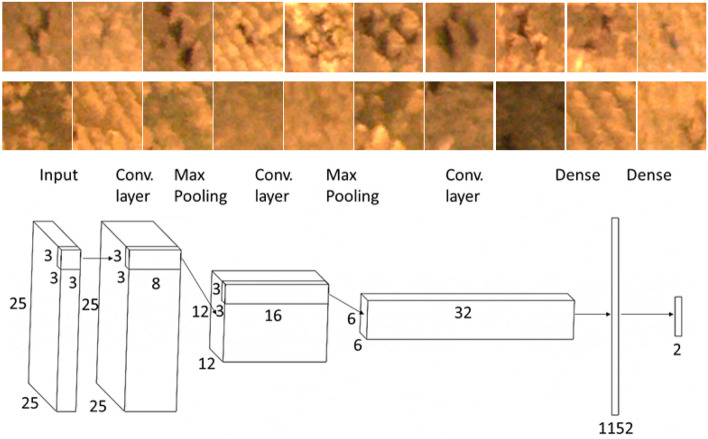
Examples of data for dots localization CNN training. Upper row – dots, bottom row – areas without dots.

The first step in dot detection was the normalization of the ROI to the length of 1000 pixels. The height of the image was calculated to maintain the height/width ratio. Normalization eliminates the change in ROI size during fish growth. The CNN was used for dot localization. The network performs the classification into two classes, dots or no-dots. The network architecture:contains five trained layers: three convolutional and two fully connected layers. The rectified linear unit (ReLU) activation function was used for convolutional layers, and the Softmax activation function, for the classification layer. Max pooling layers were used between the convolutional layers. The training was performed with the Matlab R2020b Deep learning toolbox function “trainNetwork”. The architecture of the network with training examples is shown in Fig. 7. A total of 535 examples of the area with dots and 535 examples without dots were used for CNN training. The resolution of the images was 25 × 25 pixels. These images were manually randomly selected from the images of ROIs from all SL datasets. The network was trained using 2/3 of the images. The accuracy of the CNN, tested on the testing dataset (1/3 of the images), was 99%.

**Figure 8:**
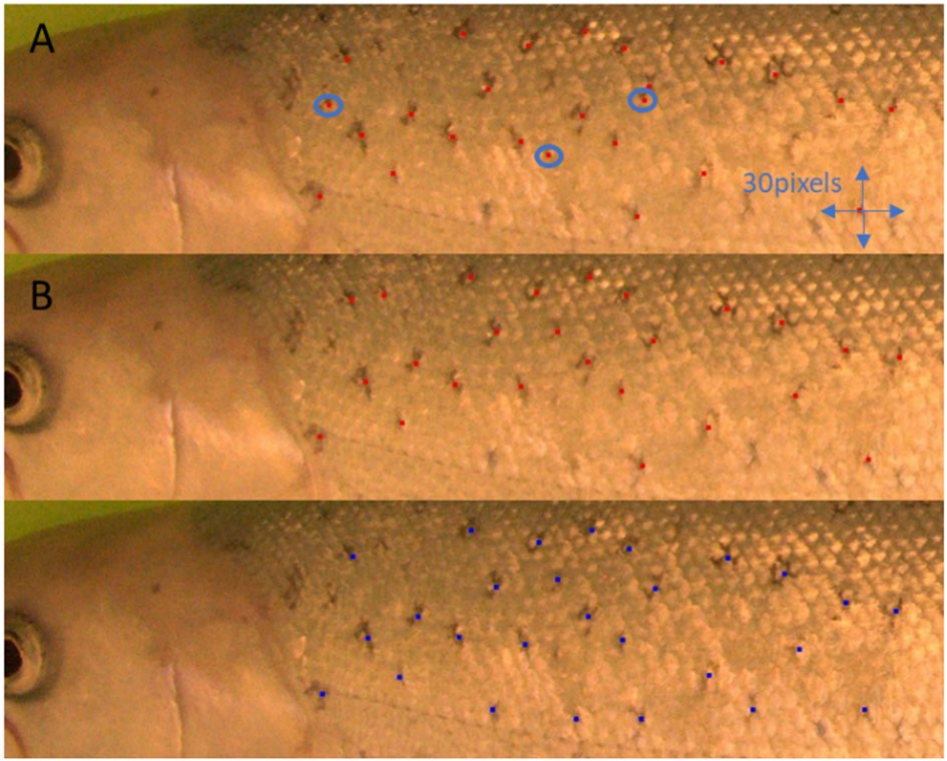
Top and middle images—example of dot detection for two (A and B) images of the same fish. The arrows indicate the pattern shift for alignment. The dots (blue circle) of the A pattern do not have close points in the B pattern, and therefore, they are not used in the representative pattern. Bottom—representative dots selected as a subset of the dots detected for all images of the same fish.

The localization of the particular dot in the ROI was performed as the classification of the sliding window (with 5 pixel step of the window in the x and y directions) over the ROI into the dot or no dot class. The sliding window was classified as dot if the dot class’ probability was higher than 30%. The dots were detected between 1/3 and 3/3 of the length of the ROI to exclude the head region (there is no dot pattern on the head). The method identifies a high number of dots, and some dots are detected more than once. Therefore, the dots’ clustering was used to cluster the dots closer than 15 pixels using the Matlab function *clusterXYpoints*. The final dots are represented as centroids of the detected clusters (see Fig. 8). The coordinates of the dots were saved as the dot pattern describing the fish.

### Short term identification

The dot pattern was detected for all images for all fish in the SS and SL1 datasets. SS + SL1 dataset was collected for the 328 fish, where the rotation and translation of the fish were applied during the data collection. One dot pattern was selected as a comparative image for each of the 328 fish. Two other dot patterns for the fish were used as the fish template. Identification was performed to compare the relative pattern with two templates for each fish (nearest neighbour classification). The distance between the dot patterns was measured as the average distance between ¾ of the points of the two compared patterns. For each dot in the first pattern, the closest dot in the second pattern (according to Euclidean distance) was determined. The distances of all dots were sorted by size, and the average distance was calculated for the first ¾ of the points of the first pattern. The last ¼ of the distances were not used, to eliminate the effects of outliers (incorrectly detected dots). Because of the possibility of the dots shifting in the x and y axes (the ROI not being correctly determined), the calculation of the distance between the two patterns was repeated for the second dot pattern shifted ± 30 pixels with a step of 5 pixels in the x and y directions. The determination of the closest point and calculation of the average distance was performed for all shifts. The minimal distance was then used as the final distance between the two patterns. The fish was then identified as the fish with the minimal final distance between the comparative pattern and one of the two template patterns. The number of dots in the pattern varied from 4 to more than 30. A heuristic to minimize the calculation time was applied for the fish comparison. The dot pattern of fish A was compared with that of fish B only if the number of dots for B was up two times lower or higher than that of A. This approach compared only similar patterns.

Two-point cloud registration methods (iterative closest point (ICP)^24^ and coherent point drift (CPD)^25^ implemented in Matlab (function *pcregisterict* and *pcregistercpd*) were tested for the matching of the dot patterns, but they achieved lower accuracy than the sliding window method.

### Representative pattern

The representative dot pattern for each fish was determined to improve the identification and reduce the time of the calculations. All dot patterns of one fish were compared, and the points appearing on 1/3 of the patterns were selected as the best representatives. The same approach used for the comparison of patterns for short-term identification was used. The pattern with the minimal average distance from all other patterns was used as the reference pattern. The best match to the other patterns of the same fish was detected based on the shift in x and y and calculating the average minimal distance. This step aligned all the dot patterns. For each point of the reference pattern, the closest points in all other patterns were detected. If the distance to the closest point was smaller than 20 pixels (it meant that the point appeared in more images of the fish) then it was selected for the representative dot pattern. The point was selected if it appeared on more than 1/3 or at least three images (for low numbers of images). See an example of a representative dot pattern in Fig. 8. The representative patterns represent detected dots for the particular fish.

### Long term identificaton

The long-term identification task involved the dot pattern stability for long-term fish cultivation. Based on the manual analysis described in the Sect. 2.7., it was determined that the automatic fish rotation compensates the different poses of the fish in the original image. Therefore, the main difference between the dot patterns caused by fish growth is in the horizontal/vertical direction and scale. No other distortions corrupted the dot pattern over the six months period (missing scales are discussed in the Discussion ). The long-term identification used the same approach as described for short-term. The only difference was that the representative patterns awere used for identification instead of the detected dot patterns and the maximal shift (used for dot alignment) was 50 pixels. The identification was made separately for all combinations of the SL datasets to test the identification for different fish ages and different periods (see Fig. 3).

## Histogram of oriented gradients (HOG) approach

**Figure 9:**
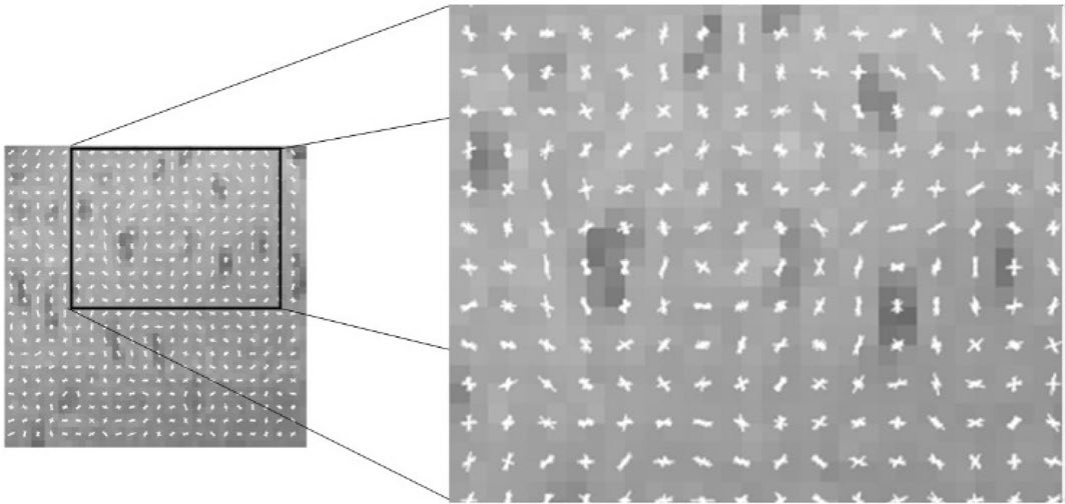
Visualization of HOG descriptor for the dots pattern. Left – normalized image of ROI (zoom of 64*64 pixels image). Right – orientation of the edges in the image coded by HOGs (zoom of left image). Produced by Matlab R2020b.

**Figure 10:**
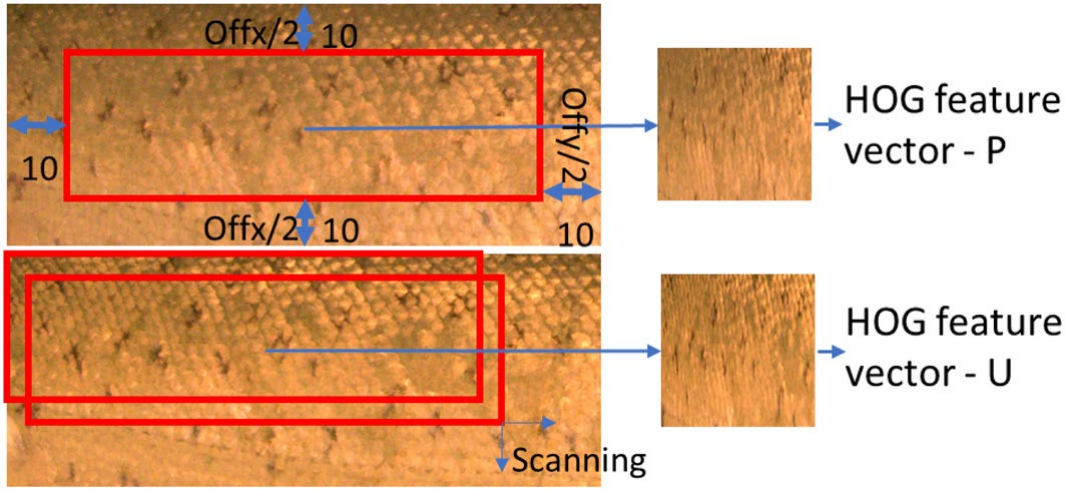
Similarity measure of two fish skin patterns using HOG feature descriptor. Upper row (P)—image of the identified fish. The subpart of the pattern is used for parametrization. The subpart is normalized to a 64 × 64 pixel image, and the HOG feature vector is calculated. Bottom row (U)—images of the unknown fish. The subpart of the pattern is selected from the image repeatedly by scanning over the image. The subpart is normalized to a 64 × 64 pixel image, and the HOG feature vector is calculated. The similarity between the P and U images is the best similarity between the P subset and one of the U subsets from the scanning.

Histogram of oriented gradients^26^ is a feature descriptor of image pattern parametrization mainly used for object detection in computer vision. The HOG feature descriptor codes the gradients of the image, which mainly represent the edges or points in the image, and therefore, it is also a good texture descriptor invariant with the illumination. The identification using HOG consisted of the following steps: (1) feature vector calculation for the pattern from the ROI of the known fish (see Fig. 9), (2) scanning of ROI for unknows fish, (3) the calculation of the best similarity between the known and unknown fish, (4) the classification of unknow fish into one of the know fish. The classification using HOG was based on the calculation of the similarity of the HOG vectors of the ROIs for both known and unknown fish. The HOG vector was first calculated for known fish (i.e., the fish with known ID). The only subset of the ROI was used for parameterization (see Fig. 10). The subset was selected as the ROI without the border areas of the size of Offx and Offy. The subset was resized to a sizeN*sizeN pixel image, and the HOG feature vector was calculated. Then, the HOG feature vector was calculated from a subpart of the ROI of the unknown fish (fish we identified). The same size of subpart as for the known fish was used. The subpart was repeatedly selected using a sliding window over the ROI of the unknown fish. The similarity (distance) of the two HOG vectors (known and unknown fish) was calculated as the norm between the two vectors. The similarity was determined as the minimal distance between the known HOG vector and all HOG vectors generated by scanning over the unknown fish ROI. The function extractHOGFeatures—Matlab R2020b was used for HOG calculation.

### Identification

The same tasks of identification were performed as for the dot pattern approach described in Section 2.5. The short-term identification was performed using the SS and SL1 datasets together. The first image of all images for the particular fish was used as the unknow image. The second and third images of all for the particular fish were used as the known images. For each fish the similarity to all other fish was calculated based on the scanning described above. The fish was identified as the most similar fish. For long-term identification, all images of the fish from one SL dataset were used as the known images and all images of the fish from another dataset were used as the unknown images. The similarity between all combinations of the images for the two fish was calculated using the scanning approach, and the best similarity was taken as the similarity between these two fish. The similarity was calculated for all fish and the two SL datasets. Each fish from first SL dataset was identified as the most similar fish from the second SL dataset.

Different settings of the parameters of HOG feature calculation were tested: the cell size, resolution of normalization image and the offset Offx and Offy. Different subregions of the ROI were also tested for HOG feature calculation. The right half, right 2/3 and right 4/3 of the ROI in the horizontal direction were tested as subregions. Only the best results and settings are described in the Results section.

## Pattern validation

**Figure 11:**
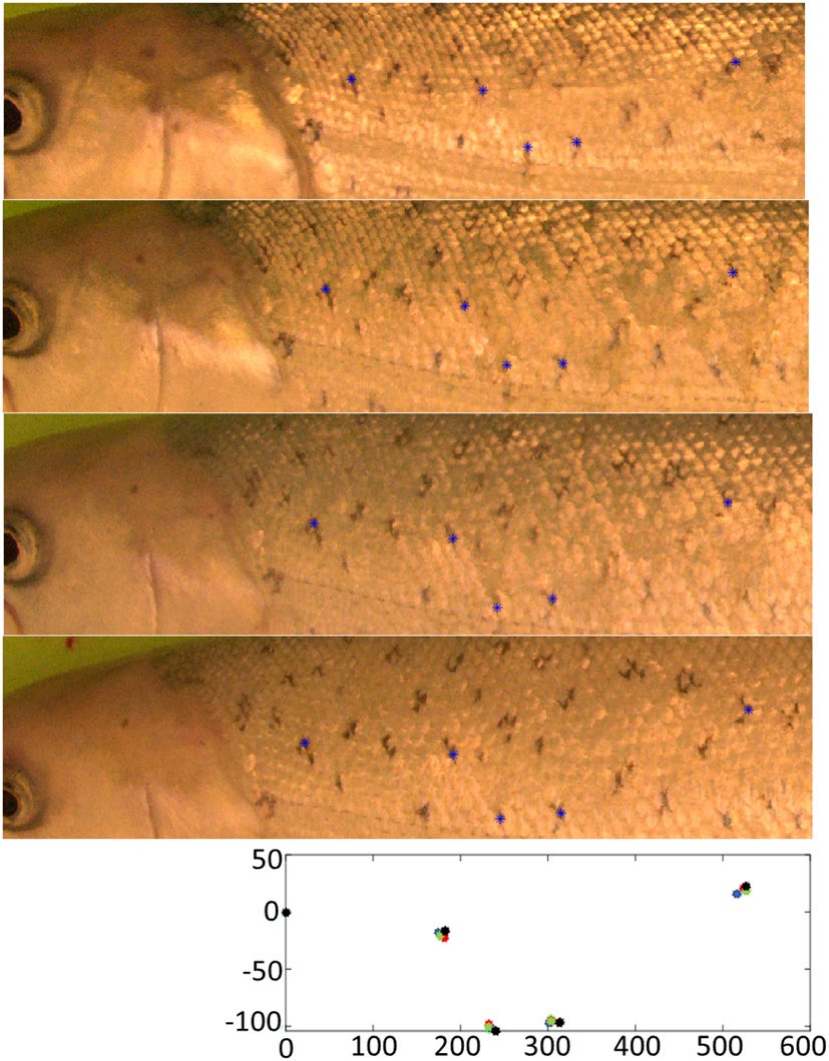
Manual localization of dots for one fish for four sessions, SL1–SL4. Blue dots were manually localized. Bottom plot shows aligned dots from SL1–SL4. Manually localized dots were used for analysis of pattern stability.

ROI localization and dot detection were performed automatically, which could influence the identification accuracy. Therefore, we performed a manual analysis of the dot pattern changes during the six months of fish growth. Five fish out of the 30 from the SL datasets were randomly selected for manual analysis. The same five dots at different places of the ROI were selected for each fish. The dots were manually localized in one image for all four SL datasets for all five fish (see Fig. 11). The x and y shift, rotation and scale were applied to the localized dots to obtain the best alignment for each fish separately. The average displacement of the five points was calculated after the alignment to analyze the pattern changes over time.

## Results

### Manual evaluation

The average displacement of the four points for all five fish was 9 pixels for the x axes and 8 pixels for the y axes. The displacement was calculated for each fish and for each point separately as the distance of the point in the first image and other images. The image resolution was 1000 pixels in the x axis and 269 (average) pixels in y axes. See the displacements of the points in Fig. 11. The displacement is partially caused by the change in the pattern (fish growth) and partially by the manual localization. The dot pattern does not have any exact shape, and the shape of the dots is mainly changed during fish cultivation. The labeler selected one exact point in the dot, which was used for the labeling. We estimate that half of the displacements were due to the labeling itself. The displacement of 5 pixels proved that the dot pattern was stable and usable for image-based identification for at least six months.

### ROI localization

The localization of the ROI is the most critical part of the identification. Manual analysis proved that the dot pattern was stable. For successful identification, the same part of the pattern has to be selected. The ROI was detected with the maximal displacement of 50 pixels in both directions. It was 5% for the x axes and approximately 12% for the y axes. The localization depended mainly on fish bending in the z axes (distance from the camera) and the change on fish proportions caused by fish growth.

### Identification

Two methods were used for the automatic identification of the individuals. Both methods used the same ROI for identification. Two tasks of identification were performed. First, the pattern uniqueness was tested using the SS + SL1 dataset. Both methods obtained 100% accuracy for identification from the 328 fish tent and aquarium data. It is proof that the pattern is unique for a high number of fish and can be used for identification. The second task was to test the stability of the pattern during fish growth. Four SL datasets were used for this task with the data of the fish in the tent and aquarium. The best results were achieved using the identification based on dot detection. The accuracy was 100% for all the combinations of all datasets for the tent data. The accuracy for aquarium data was also 100% for all combinations except for the identification for the SL1/SL3 (accuracy 96.7%) and SL1/SL4 (accuracy 70%) datasets, representing identification after 4 and 6 months of growth, respectively. The results of the method based on HOG are summarized in Table 1. The best results were obtained using the following settings: normalized image size, 64*64; offset, 10*10; cell size, 2; ¾ROI for the SL1/SL4 dataset and 2/3ROI for all other combinations of datasets.

**Table 1:** The accuracy of automatic identification using hog descriptor of the pattern and dot approach. The accuracy in % is shown for the combinations of the SL datasets.

HOG-based identification										Dot-based identification				
Tent data					Aquarium data					Aquarium data				
Session	SL1	SL2	SL3	SL4	Session	SL1	SL2	SL3	SL4	Session	SL1	SL2	SL3	SL4
SL1	100	100	83.3	36.6	SL1	100	83.3	73.3	70	SL1	100	100	96.7	70
SL2		100	100	53.3	SL2		100	76.6	66.6	SL2		100	100	100
SL3			100	93.3	SL3			100	60	SL3			100	100
SL4				100	SL4				100	SL4				100

It can be seen that both methods obtained an accuracy of 100% for the datasets taken over two months. The accuracy for the other combinations (more distant in time) decreased and was correlated with the time interval between the data collections for HOG approach.

The identification of one individual into 30 classes using the dot approach took 0.8 s (CPU – Intel i5-6300CPU). The whole code was implemented in Matlab R2020b without any optimization or precalculated representations of the patterns.

## Discussion

This study is the most extensive study of automatic individual fish identification based on fish skin patterns. The study was large because 328 fish were used to test the feasibility of using the lateral skin dot pattern of Atlantic salmon for individual identification and a 6 month period of fish growth was used to study the pattern stability during fish cultivation. Previous studies had only identified a maximum of 30 fish with semi-automated methods^18^ and five fish with fully automated methods^19^, and there were none using fully automated methods for long-term identification. This study is the first in which images of fish out of the water (i.e., in the tent) and underwater (i.e., in the aquarium) were used for long-term automatic identification. All other studies used images of fish taken out of water only.

Individual fish identification is widely used in aquaculture research where it is necessary to study individual behaviour, growth or fish states according to environmental changes or feeding strategies. The benefit of remote fish identification based on appearance only is evident for research purposes because of its non-invasiveness and the possibility of identifying fish too small to be tagged^10^. The need for individual fish identification is also becoming more critical for aquaculture production with the automation of production monitoring and control. It is not yet widely used, mainly because of the limitations of the existing invasive tagging methods. The fish must be caught to be tagged, which is time consuming and often impossible in high-density fish cultivation (200 000 fish in the cage) in the sea cages^27^. The price of such a large number of tags is enormous for fish farmers. Another limitation is tag reading underwater and the need for tag removal before the fish are delivered to the market. The remote individual identification method, which could be used in the tanks or sea cages without the need to manipulate the fish could enable new methods of monitoring fish growth and state. Individual identification can be used to improve biomass estimation, fish sorting based on the signs of disease or individual growth. The approach of individual identification based on appearance must be fully automatic, accurate for long-term fish cultivation and able to work under production conditions to be useful for the aquaculture industry. This study examined the possibility of the automatic individual identification of Atlantic salmon over 6 months of cultivation.

Two datasets were recorded for the study: a tent dataset representing high quality images of the fish out of the water; an aquarium dataset representing lower quality data due to the light scattering by the water and varying illumination in each recording session.

Two methods were used for fully automatized individual fish identification: a dot localization method and HOG feature-based method. The accuracy of both methods for the short-term identification of 328 individuals for tent and aquarium images was 100%. The accuracy of long-term identification with the tent data was 100% for six months and that with the aquarium data was 70% (100% for four months), for the dot localization method.

Originally, more texture descriptors (local binary patterns, scale invariant feature tranform), and an approach completely based on CNN (end-to-end) were tested for fish identification. The best accuracy was obtained using the HOG descriptor. Because the HOG-based long-term identification did not reach 100% accuracy, the specific dot localization method was developed, which shows better accuracy for most identification tasks.

Different aproaches were used for dot alignement for dot based identificaiton. The methods for point cloud registration^24,25^ obtained lower accuracy then the simple but effective method of using poitn shift and scale. The lower accuracy was caused by the incorrect alignment of the dot patterns with low numbers of dots. The methods expect that the point clouds used for registration contain the inliers and outliers, which is not not the case with the low numbers of dots patterns.

The short-term identification of the 328 fish proved that the fish skin’s melanophore dot pattern is unique for distinguishing all the fish without any error. The fish rotation, translation and illumination changes did not cause any misidentification. The identification was performed for all the SS and SL datasets, and the fish growth did not influence the accuracy. The dot pattern had better contrast for older fish than for the fish in the first session. The result cannot be directly generalized for more fish but the differences in the numbers of dots and the positions are high, which is a good assumption for the identification of a higher number of fish.

The long-term stability of the pattern is critical for accurate identification in aquaculture production. The fish are usually cultivated for several months/years and should be identifiable for the whole cultivation period. The four SL datasets were recorded to cover the fish growth, which could influence the skin dot patterns. Six months of data collection started at the fish age of 5 months and finished at the age of 11 months. Visual inspection of the data recorded at the different sessions showed that the dot pattern is more visible on older fish. Especially, the contrast between the dots and the surrounding skin is higher. This is consistent with the observations of Stien^18^. During the 6-month period the fish length changed 1.6 times and the fish height changed 1.9 times, on average. Manual verification of the pattern stability was therefore performed to analyze pattern changes. The dot displacement was estimated to be 9 pixels for the image of 1000 pixels in width—0.9% of the image’s width. The translation, rotation and scale were applied to align the patterns for all sessions. The result of the analysis was that the dot pattern was stable based on the dot’s position for 6 months and could be used for long-term identification.

Both methods (HOG and dots) were used to test the fish’s identification for 6 months. All possible combinations of SL datasets were used for testing. The combination SL1/SL2 represents the identification after 2 months of cultivation. The combination SL1/SL4 represents the identification after 6 months of cultivation. The dot-based identification method achieved 100% accuracy for all combinations using the tent data. Using the aquarium data, the accuracy dropped for the combination SL1/SL3 and the SL1/SL4 dataset, representing 4- and 6-month differences, respectively. The main reason for the lower accuracy with the aquarium data could be the lower data quality and high difference in the illumination in each session. The main difference was between the sessions SL3 and SL4. The accuracy of identification using the HOG approach was the same or lower than that with the dot approach for all combinations. For the tent data, the HOG approach could correctly (except SL3/SL4 – accuracy 93.3%) identify all fish in the 2-month data collections. The accuracy for the 4 months period was 83.3% and 53.3%. The accuracy for the 6 months period was only 36.6% only. The accuracy with the aquarium datasets was lower than that with the tent data. The average accuracy for 2 months was 73.3%; for 4 months, it was 70%; and for 6 months, it was 70%. The HOG approach’s lower accuracy is explained by coding not only the dots but also the reflections and missing scales on the fish’s bodies. The dot approach successfully eliminates these problems. The long-term identification showed that the dot approach could identify all fish without any errors over a period of 6 months using high quality (tent) data. The 100% accuracy could be achieved for lower quality data (aquarium) for a 4 month period. The HOG approach could correctly identify all fish in 2 months with high quality data. Using the lower quality data, the identification for all periods was on average 71%. Automatic long-term fish identification (for at least 6 months) based on appearance is possible using the dot approach with high quality data. The HOG approach can also be used but mainly for 2 months periods. Both methods’ results can be improved by updating the fish images that represent the individual fish. Because of the 100% accuracy for the 2-month period (SL1/SL2, SL2/SL3 and SL3/SL4), the old images of each fish can be substituted by the new images after the identification. This approach will update the representative images for the growing fish and will increase the identification accuracy during fish growth. The accuracy for the tent data for the 6-month period would be 93.3% with the HOG approach using the image update method. This approach expects that the system detects all fish during the period of 2 months.

The HOG based approach uses image with resolution of 64 × 64 pixels for identification. Therefore, there is high probability that the method will work under real conditions where the quality of the fish images will be decreased by fish movement, changing light conditions and fish overlaps. The advantage of the HOG approach is the possibility of using the method for the parametrization of the different skin patterns of the fish. The method was successfully used to identify the ornamental fish Sumatra barb (*Puntigrus tetrazona*) with the stripe pattern^28^. The dot approach can only be used for the fish with the dot pattern and the CNN for dot detection must be trained for particular species.

The HOG approach is also sensitive to the localization of the ROI. A shift in the ROI highly influences the identification accuracy. This was observed during the experiments with the selection of the best ROI for identification. Finally, the upper left part of the fish was selected because it contains the dot pattern (there are just few dots in the left bottom part) and does not deform with fish movement. The pattern on the right (tail part) part of the fish deforms due to the tail bending, which is natural during fish swimming.

The study proved that automatic individual fish identification based on dot patterns for Atlantic salmon is possible. The limitation of the current approach is that the images of the immobilized fish were used. The approach is directly useful in all studies where the fish are caught and sampled. It can be used as a substitute for the tagging method. The main potential of the method is for remote identification in aquaculture production. To apply the method inside the tanks and sea cages, high-quality data capture for the swimming fish must be achieved. This can be performed using high resolution cameras and deep learning methods, as shown by Schellewald^29^. The usability of the automatic identification method under the real conditions is also influenced by the method’s speed. The speed is not too critical. The real-world scenario of identification is that the system captures images of the fish while they are swimming around and performs the identification. The speed of identification into 30 classes was 0.8 s without any optimization. The time needed for identification of one individual in the out-of-water scenario depend mainly on the time of fish imaging. The time is from 3–10 min including fish immobilization. The HOG-based approach is general for any species with the visible pattern. Therefore, the method can be used also for laboratory and wild species. In general, the more structured (strong edges or isolated dost) pattern on the fish body the better results of the identification. The method can be also used for field experiments because the only condition of successful identification is to take good quality pictures of the fish.

The comparison of the results of the study with the state-of-the-art is complicated because of low number of studies in this field. The standard method used for fish identification is tagging. The accuracy of the developed approach and tagging is the same for 30 fish for the period of 6 months and for 328 fish for short-term identification. The main advantage of the dot approach is the non-invasiveness. The main disadvantage is that pictures of the fish have to be taken. Once the image-based identification is implemented in the tank/sea cage without the need for catching the fish, the advantage of the approach will be much higher. All studies of individual identification based on the skin pattern use low numbers of fish, are performed manually and use images of the fish out of water. The only study where a fully automatic approach was developed is that of Al-Jubouri^19^. They used a total of 50 images (10 per fish) of five zebrafish in an aquarium under controlled illumination to perform short-term identification. The reported accuracy was 99%. The dot based short-term identification of 328 individuals using aquarium data introduced in this study has a higher number of fish, greater variability in the data collection conditions and better identification accuracy. For the long-term identification, there is no study reporting automatic fish individual identification.

Future research will be focused on the implementation of image-based individual identification under real aquaculture production conditions to harness the potential of the approach. The possibility of identifying the fish without visible skin patterns will be tested to cover more species important in aquaculture.

## Conclusion

In this paper, we tested the possibility of short- and long-term automatic fish individual identification based on skin pattern for Atlantic salmon. We developed a new fully automated approach for dot-based individual identification, with an accuracy of 100% the short-term identification of for 328 individuals and 100% for long-term (6 months) identification of 30 fish. The approach was tested for fish out of the water and fish in the aquarium to approximate real conditions. The approach can be used as a substitute for identification with invasive tagging. It can be used for any species with the dot pattern. The more general HOG-based identification was also tested and shown to have loweraccuracy. The HOG-based identification can be used for fish species with patterns different from dots. Future work will be focused on the adaptation of the approach to tank/cage real conditions. Image-based fish identification under real aquaculture conditions could open new possibilities for fish maintenance and treatment.

## Data Availability

Data will be made available upon request through our data management system bioWES.

## Code availability

The source code for Matlab environment for dot-based fish identification is available upon reguest.
